# The Improvement of Growth Parameters and Intercepted Photosynthetically Active Radiation in Pea Varieties as Influenced by Nitrogen Fertilization

**DOI:** 10.3390/plants14223450

**Published:** 2025-11-11

**Authors:** Daiva Janusauskaite

**Affiliations:** Department of Plant Nutrition and Agroecology, Institute of Agriculture, Lithuanian Research Centre for Agriculture and Forestry, Instituto 1, Akademija, LT-58344 Kėdainiai, Lithuania; daiva.janusauskaite@lammc.lt

**Keywords:** aboveground dry mass, fertilization, intercepted PAR, leaves area index, *Pisum sativum* L., varieties

## Abstract

The field experiment aimed to evaluate the effect of different nitrogen rates on accumulation of aboveground dry mass (AGDM), leaves area index (LAI), and intercepted photosynthetically active radiation (iPAR) of pea (*Pisum sativum* L.) varieties. The experiment was arranged in a factorial randomized block design consisting of three levels of the first factor (variety) and seven levels of the second factor (NPK fertilization treatments were used: (1) NPK 0:0:0 (control), (2) NPK 0:40:80, (3) NPK 15:40:80, (4) NPK 30:40:80, (5) NPK 45:40:80, (6) NPK 15 + 15:40:80, (7) NPK 60:40:80). The growth indicators (LAI and AGDM) and iPAR were assessed three times during the growing season. Nitrogen fertilization positively influenced LAI, but significant differences in LAI were found only under splitted N30 (N15 + N15), N45, and N60 applications, compared to the treatment N0 P40K80. In the dry 2015 and the optimal moisture 2016, N30, N45, and N60 rates significantly increased AGDM. The influence of fertilization on iPAR varied between experimental years, and it was strongest in the dry 2015, when applying N15 + 15 and N60 fertilization significantly increased iPAR, compared to the control. According to LAI and iPAR data, pea varieties were ranked in descending order: Simona, Ieva DS, and Respect. LAI significantly (*p* ≤ 0.01) correlated with AGDM and iPAR, but the relationship weakened as peas reached later growth stages. These results provide valuable knowledge, and it will be useful for researchers in developing new cultivation methodologies to achieve higher semi-leafless pea productivity by applying different combinations of nutrition and new varieties.

## 1. Introduction

Leaf area is an important indicator of plant productivity as it is closely related to photosynthetic capacity and strongly influences photosynthetic rate [1,2]. The optimal leaf area determines the maximum amount of absorbed solar photo-active radiation [3]. The dynamics of leaf area index (LAI) formation depend on many factors—plant genetic characteristics, water regime, light, temperature, spread of plant pathogens, and agrotechnology [4,5,6]. However, nutritional conditions have the most tangible influence on this indicator of plant productivity [7,8].

Crop development and utilization of genetically encoded productivity depend on the ability of foliage to intercept solar radiation. Photosynthetically active radiation (PAR) interception is a crucial component in analyzing crop growth and assessing yield [1]. PAR is an important factor influencing dry matter production, nutrient uptake, and crop development and growth duration. The efficiency of radiation conversion into dry matter depends on plant characteristics and environmental factors [9,10]. The growth, development, and productivity of pea (*Pisum sativum* L.) crops are significantly influenced not only by temperature and moisture regimes, but also by the intercepted photosynthetically active radiation [11]. In this regard, it is of great importance to evaluate the interaction of peas with the dominant radiation regime at different growth stages throughout the vegetation period, which would help to better understand the role of intercepted PAR in the production of assimilates. PAR is required for plant growth and development and is also closely related to the variation in leaf area index and biomass production [12,13]. Information on PAR and its interception in foliage is crucial for explaining causal relationships between environmental factors and variation in pea productivity and resource efficiencies. Studies on corn and soybeans have shown that the amount of light reflected by plant leaves depends on the water movement and physiological processes occurring in the leaves, and on the microclimate in the crop [14]. Light interception by plant foliage is complicated and depends on the orientation of the plant row relative to the sun, leaf architecture, leaf optical properties, and the angle of elevation of the sun [12,15,16].

The genetic diversity of peas is very high; there are varieties of winter and summer peas. According to the ripening time, there are very early and late ripening varieties; according to the morphological composition, there are varieties with leaves and semi-leafless [10]. Comparisons of the yield of leafy and semi-leafless pea genotypes can be found in the literature, but in many cases contradictory results have been obtained under different soil and climatic conditions [10,17,18]. Other studies have shown that fertilizers supply affects iPAR in peas [8,19,20]. There is a lack of knowledge about the direct effect of pea fertilization on iPAR [4,9]. Therefore, it is important to determine the PAR absorbed by semi-leafless varieties and the differences in dry matter production during the vegetation period. It should be noted that the development of agrotechnology for semi-leafless peas is not yet complete, and many questions need to be ascertained. The study aimed to determine the influence of nutritional conditions on leaf area index, dry matter production, and absorption of photosynthetically active radiation at different growth stages and to evaluate the differences in these parameters between three pea varieties. We hope that this study will identify optimal nutritional conditions for peas, enabling them to achieve the highest growth indicators and iPAR in the crop, and establish differences between varieties.

## 2. Results

### 2.1. Leaf Area Index (LAI)

Three-factor analysis of variance showed that in all years of the study, the growth stage (GS) (factor A), variety (factor B), and fertilization (factor C) in all cases significantly influenced LAI values at the 99% probability level (*p* ≤ 0.01) (Table 1). GS was the main factor that explained 12.7–61.2% of the total variability in LAI. Variety determined 13.3–29.8% differences between treatments. Fertilization impact was significant (*p* ≤ 0.01), but the weakest (only 0.6–11.4% contribution to total variance in LAI).

**Figure 1:**
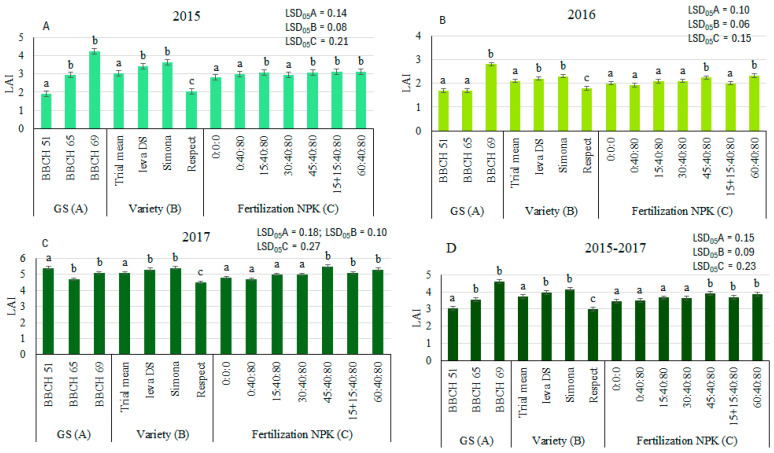
The effect of growth stage (GS), variety, and fertilization on LAI of pea, 2015 (**A**), 2016 (**B**), 2017 (**C**), and average 2015–2017 (**D**). Different letters in bars denote the significant differences (at *p* ≤ 0.05 according to LSD) between treatments.

In 2015 and 2016, LAI increased consistently throughout the vegetation period, and compared with LAI at BBCH 51, LAI values significantly increased by 1.0–2.3 (or 52–120%) (Figure 1). The opposite dynamics of LAI occurred in 2017, which was characterized by an uneven distribution of precipitation (May with almost no precipitation, and July with heavy rains). Under the mentioned conditions, the highest LAI (5.4) was recorded in BBCH 51, and LAI decreased by 0.7 and 0.2 (or 12 and 4%) in subsequent stages.

According to LAI, pea varieties were ranked in descending order: Simona, Ieva DS, and Respect. Compared to the trial mean, LAI for the variety Simona significantly exceeded the trial mean by 6.5–19.4%, Ieva DS by 4.3–13.2%. Meanwhile, LAI for the variety Respect was significantly lower by 11.1–33.2% than the trial mean in all experimental years.

Nitrogen (N) fertilization positively influenced LAI, increasing it on average by 1.7–4.6%, 4.7–19.7%, and 6.6–17.7%, respectively, in 2015, 2016, and 2017, compared to the treatment N0P40K80 (Figure 1). The lowest rates of N15 and N30 had no significant effect on LAI in most cases. LAI values significantly increased under N45 and N60 fertilization, by 2.3–17.7% and 4.6–19.7%, respectively, in comparison with the N0P40K80 treatment. The splitting of N30 (N15 + N15) significantly increased LAI by 3.0% and 9.4%, compared with N0P40K80, in 2015 and 2017, respectively.

Compared to the control without fertilizer N0P0K0, the NPK combination increased LAI by an average of 4.6–11.7%, 2.0–16.7%, and 3.3–14.1% in 2015, 2016, and 2017, respectively.

### 2.2. Aboveground Dry Mass (AGDM)

According to a three-way ANOVA, aboveground dry mass (AGDM) of pea was significantly influenced by GS (factor A), variety (factor B), and fertilization (factor C) at the 99% probability level (*p* ≤ 0.01), with one exception in 2017 (Table 1). GS was responsible for the greatest part (47.4–75.5%) of the total variability of AGDM data. Variety determined 2.7–14.1% of AGDM variation. Fertilization explained only 0.8–4.7% of AGDM differences.

**Figure 2:**
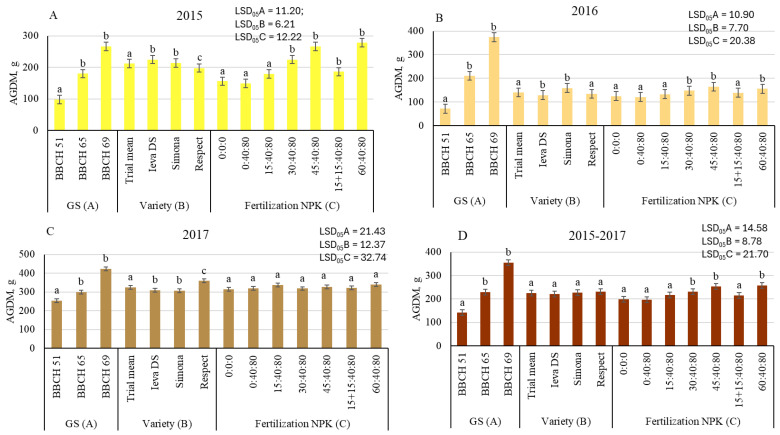
The effect of growth stage (GS), variety, and fertilization on aboveground dry mass (AGDM) of pea, 2015 (**A**), 2016 (**B**), 2017 (**C**), and average 2015–2017 (**D**)). Different letters in bars denote the significant differences (at *p* ≤ 0.05 according to LSD) between treatments.

Compared to BBCH 51, AGDM was significantly higher at later GS. The differences in AGDM data ranged from 18 to 194%, and from 67 to 423%, respectively, at BBCH 65 and BBCH 69 GS, compared to those found at BBCH 51 (Figure 2).

According to AGDM data, it can be concluded that different varieties of peas responded differently to moisture conditions during the season. Compared to the trial mean, the lowest AGDM (−6.6%) was of the variety Respect, which reacted more sensitively than the other two varieties to drought conditions in 2015, which are described as dry according to the hydrothermal coefficient (HTC 1.0). Meanwhile, in 2017, when HTC was 1.4, the variety Respect demonstrated the highest AGDM, and compared with the trial mean, significantly surpassed it by 10.5%. The AGDM values of the varieties Ieva DS and Simona were close to the trial mean in most cases.

Nitrogen (N) efficiency on AGDM accumulation was different in the experimental years (Figure 2). In the dry 2015 and the optimal moisture 2016, N30, N45, and N60 rates significantly increased AGDM (by 44.2–78.2% and by 18.1–31.3%), but the differences were not significant in all cases. The lowest N fertilizer efficiency was in 2017, when AGDM was 3.8–7.9% higher compared to the control, but the differences were insignificant in all cases. The splitting the N30 rate into two doses (N15 + 15) did not result in a significant increase in AGDM, compared to both the control and N30.

### 2.3. Intercepted Photosynthetically Active Radiation (iPAR)

A three-way ANOVA showed that GS (factor A) had a significant (*p* ≤ 0.01) effect on intercepted photosynthetically active radiation (iPAR) and was the main factor determined 3.6–72.4% of the iPAR data variation (Table 1). The variety (factor B) was responsible for 8.4–22.4% of the iPAR differences between treatments. Fertilization (factor C) explained the least part (2.1–6.9%) of iPAR variation.

**Figure 3:**
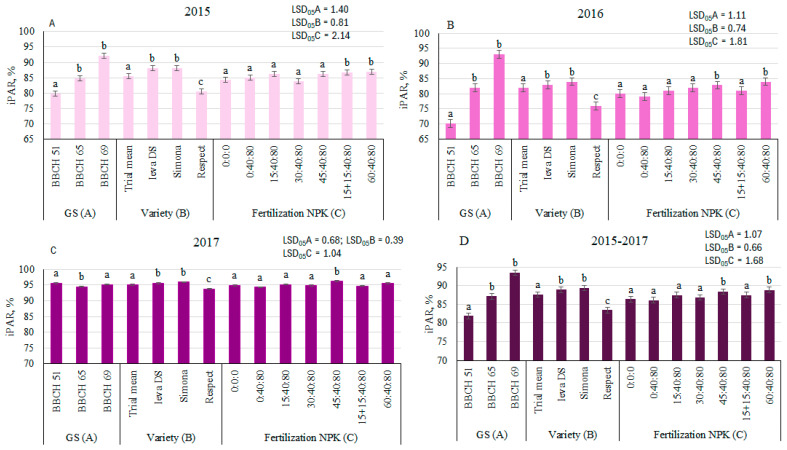
The effect of growth stage (GS), variety, and fertilization on iPAR of pea, 2015 (**A**), 2016 (**B**), 2017 (**C**), and average 2015–2017 (**D**). Different letters in bars denote the significant differences (at *p* ≤ 0.05 according to LSD) between treatments.

It was found that in 2015 and 2016, radiation consistently and significantly increased at BBCH 65 and BBCH 69, compared to that found at BBCH 51; the differences in different GS were 6.5, 15.5%, and 16.8, 32.5%, respectively. In 2017, the date of iPAR was irregular and differed from the trends of other experimental years. The iPAR date was similar in all GS (Figure 3).

The varieties Ieva DS and Simona demonstrated the highest iPAR values in 2015 and 2016, and, compared with the trial mean, significantly surpassed it by 2.9 and 2.5%, and 3.0 and 4.1%, respectively. iPAR for the variety Respect was significantly lower (by 1.4–6.6%) than the trial mean in all experimental years.

The influence of fertilization on iPAR varied between experimental years. In 2015, iPAR significantly increased with N15 + N15 and N60 fertilization by 2.0 and 2.4%, respectively, compared to treatment N0P40K80 (Figure 3). The highest iPAR was determined with fertilization with the highest fertilizer rates N60P40K80. In 2016, compared to the N0P40K80, the application of N45 and N60 significantly increased iPAR by 6.5 and 8.4%, respectively. Meanwhile, in 2017, only the application of N45 showed a significant increase in iPAR (by 2.1%); in other fertilization treatments, the differences were small and insignificant, compared with the control.

### 2.4. Correlation Between Seed Yield and the Investigated Characteristics of Pea

Correlation was established between the studied indicators (Table 2). Data averaged across varieties showed that LAI significantly (*p* ≤ 0.01) and positively correlated with AGDM and iPAR in all cases. It was found that the strongest relationship was in the earlier GS (BBCH 51), while as the peas flowered or finished flowering (BBCH 65 and BBCH 69), the relationship weakened but remained statistically significant.

The relationship between SY, LAI, and AGDM, regardless of the GS during the measurement, was positive and significant (*p* ≤ 0.05, *p* ≤ 0.01) in all varieties of peas (Table 3). SY relation with iPAR was determined only in the varieties Simona and Respect (*p* ≤ 0.01), while the yield of the variety Ieva DS did not correlate with iPAR. Protein content (PC) correlated with LAI, AGDM, and iPAR in most cases in all varieties (*p* ≤ 0.05, *p* ≤ 0.01). However, the correlation of PC with the measured parameters in the Respect variety was negative. The strongest correlation between TSW and LAI, AGDM and iPAR was found for the variety Respect, to be positive and significant in all cases (*p* ≤ 0.01). Data, averaged across varieties, revealed that SY correlated positively and significantly (*p* ≤ 0.01) with measured parameters. PC significantly correlated only with LAI at different GS.

### 2.5. Correlation and Linear Regression Between iPAR and LAI, AGDM and iPAR at Different Growth Stages in Three Pea Varieties

The relationship between iPAR and LAI, and between iPAR and AGDM was described by a linear equation (Table 4). The correlation was significant in all cases at *p* ≤ 0.05 or at *p* ≤ 0.01. Based on regression equations, it was found that when LAI increased, values of iPAR also increased. The relationship between iPAR and AGDM shows the same pattern—increasing iPAR in all cases increased AGDM. Data averaged across varieties showed that at the earlier GS (BBCH 51), the relationship between the mentioned indicators was strongest (0.854 ** and 0.658 **), but the relationship weakened as the plant flowered or finished flowering. Determination coefficient (R^2^) indicated that R2 values indicated that about 85,4, 80,8, and 29.4% variations in iPAR data at BBCH51, BBCH 65, and BBCH 69, respectively, could be explained by the variation in LAI, whereas iPAR data influenced 65,8, 36,6, and 13.4%, respectively, in AGDM variations.

The multiple linear regression model revealed that about 39.3%, 28.7%, and 55.5% of the SY variation could be explained by variations in LAI, iPAR, and AGDM values at BBCH 51, BBCH 65, and BBCH 69, respectively. The relationship between tested indices was moderate to strong (R = 0.536–0.745) (Table 5).

We estimated the relation between LAI, AGDM, and meteorological factors such as precipitation and accumulated growing degree days >10 °C. A significant correlation was found and was strong in most cases (Table 5). The precipitation and accumulated growing degree days >10 °C influenced LAI and AGDM data variation by 29.1–83.5% and 52.0–90.8%, respectively.

### 2.6. Agronomic Nitrogen Use Efficiency (NUE) in Pea

**Figure 4:**
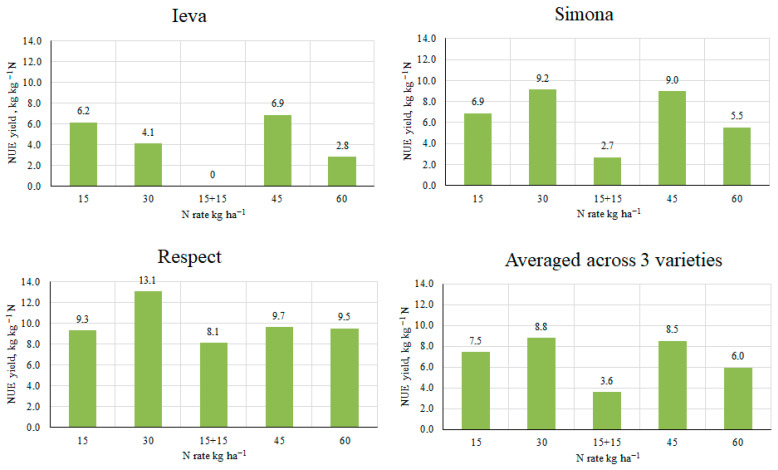
NUE _yield_ as affected by N rate (2015–2017).

The highest agronomic NUE _yield_ (from 8.1 kg to 13.1 kg of grain per kilogram of fertilizer active ingredient) was in the Respect variety and decreased in the following sequence: Respect → Simona → Ieva DS. On the P40K80 background, averaged across varieties, the efficiency of N fertilizers varied from 6.0 kg to 8.8 kg kg^−1^ N (Figure 4). Applying the N30 twice as N15 + N15, the efficiency of N fertilizers decreased by 65%. Data averaged across varieties showed that the highest values of NUE _yield_ (8.8 and 8.5 kg kg^−1^ N) were established by applying N30 and N45, respectively.

**Figure 5:**
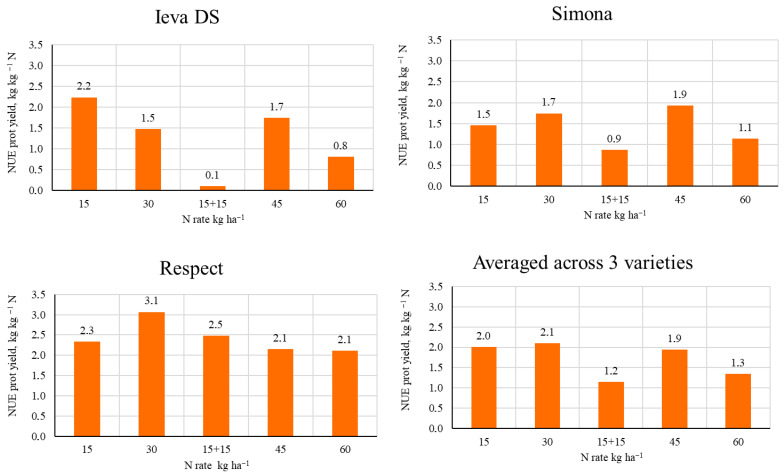
NUE _protein yield_ as affected by N rate (2015–2017).

The varieties differed in NUE _prot yield_, values ranging from 0.1 to 2.2, 0.9–1.9 and 2.1–3.1 kg kg^−1^ N for Ieva DS, Simona, and Respect, respectively (Figure 5). The highest value of NUE _prot yield_ (3.1 kg kg^−1^ N) was established by applying N30 for the variety Respect. Ieva DS and Simona achieved the highest NUE _prot yield_ values when N15 and N45 were fertilized, respectively (2.2 and 1.9 kg/kg N). The data averaged across varieties revealed that the highest values of NUE _prot yield_ (2.0 and 2.1 kg kg^−1^ N) were achieved by applying N15 and N30. Splitting the N30 rate in two reduced the NUE _prot yield_ by 42.9%, in comparison with N30.

We established the relationship between NUE _yield_, NUE _protein yield_ and LAI, AGDM, and iPAR. It was found that the correlation for varieties Ieva DS and Respect was not significant in all cases. Significant relationships were only for the variety Simona. NUE _yield_ and NUE _protein yield_ of variety Simona correlated with LAI, and the strongest correlation was with LAI3 at BBCH 69, respectively, r = 0.741 * and r = 0.729 *.

## 3. Discussion

The main factor in biological yield is the amount of light intercepted by the crop during the season, which depends on many conditions [21]. Photosynthetically active radiation (PAR) is the basic source of energy for biomass formation [13]. The absorption of radiation and its utilization efficiency of a plant may vary depending on the radiation distribution within the foliage, which is greatly influenced by canopy density, plant architecture, solar shine angle, and other factors [21,22,23]. There is data from experiments with legumes that support a positive and direct relationship between biomass production and the rate of absorption of PAR [19,24]. Later studies revealed that the intercepted PAR by a crop canopy is primarily governed by the leaf area index (LAI), independent of its canopy architecture [2,19,25]. Leaf area enlargement determines the rate of vegetative development of the plant and increases PAR retention, thus contributing to increased productivity [12,15,20]. Therefore, it is important to know which agrotechnical measures are suitable for plant LAI enlargement. These measures can be various (irrigation, balanced fertilization, plant density, tillage methods, residue management [6,11,16,22,26,27]).

The results of this study indicated that fertilization governed 1.2–10.5 percent of the total variability of pea LAI data in different years. The experimental years differed in humidity and temperature regime, based on the hydrothermal coefficient (HTK) in 2015, 2016, and 2017 were 1.0, 1.3, and 1.4, respectively. We found that LAI was highly and significantly correlated with precipitation and accumulated growing degree days >10 °C, at BBCH 51, BBCH 65, and BBCH 69, respectively, R = 0.910 **, R = 0.902 **, and R = 0.521 ** (Table 5). Thus, weather conditions in different years determined the influence of fertilizers on the variation in LAI values. Our findings are in line with previous results [28], stating that with a high total precipitation, the LAI value was almost twice as high as in the dry years. Semi-leafless pea varieties are more resistant to adverse conditions, especially during drought, and have a higher water use efficiency that is more favourable for yield formation [29]. Under non-optimum weather conditions at early growth stages, semi-leafless varieties are more productive and give better quality seeds than leafed varieties [17]. The influence of environmental conditions on LAI is presented in many studies with various legume crops—chickpea [30], grass pea [1], lentil [31,32], black gram [20], and pea [27,33,34]. Barenjee et al. [20] reported, on the contrary, that foliar fertilization, irrespective of years, equally increased LAI. We found that applying N45 and N60 rates significantly increased LAI, compared with the control. Our findings are consistent with those of Uddin et al. [35], who also determined a positive and significant impact of NPK fertilizer (N40P20K25) on LAI and seed yield. Al-Majdi et al. [36] reported that LAI increased by increasing the N fertilizer rate to 70 kg/ha. However, Yeremko et al. [37] stated that LAI significantly increased from lower rates, N15 and N30.

In an experiment with leafy and semi-leafy pea varieties, it was found that the semi-leafy variety retained a higher LAI for longer, even until the beginning of the maturity stage, possibly due to its slow-growing tendrils [33]. Semi-leafless pea varieties, which only have a stipule near the tendril, respond differently to fertilization and, unlike other plants, grow tendrils that also participate in the photosynthesis process. We found that LAI significantly correlated with seed yield at BBCH 51, BBCH 65, and BBCH 69, respectively, for variety Respect r = 0.792 **, 0.749 **, 0.702 **, for variety Simona r = 0.556 **, 0.620 **, 0.716 **, for variety Ieva DS r = 0.202, 0.467 *, and 0.692 ** (Table 3). Data from other studies also indicate a strong relationship between LAI and seed yield [21,37,38,39].

We found differences in LAI between varieties. The Simona variety had significantly the lowest LAI. The data from previous studies are in line with ours, where it is also stated that LAI differs significantly between pea varieties [21,33,39].

In the current study, significant differences in AGDM values between fertilization treatments were found only in the first two years of the experiment. The application of N30–60 rates significantly increased AGDM, compared with the control. Crop biomass accumulation is well predicted by intercepted PAR [11,12,16]. Light absorption and its direct influence on growth are important factors in plant life [11]. In the cultivation of legumes, important indicators are the amount of photosynthetically active radiation received and the efficiency of solar radiation utilization by the plant canopy [11,32]. We found that AGDM significantly correlated with iPAR at BBCH 51, BBCH 65, and BBCH 69, across varieties, respectively, r = 0.803 **, r = 0.612 **, and r = 0.370 **. Czerednik [21] indicates a positive and strong correlation between AGDM and iPAR in peas. We also found a positive relationship between these indicators, but the strength of the relationship weakened as peas developed and reached a later growth stage (Table 2). Our findings are in line with the results of an experiment with grass pea, where it was found that approximately 83.15 to 96.69% of accumulated AGDM is influenced by iPAR variation at different developmental stages [20]. ur Rahman et al. [31] observed that AGDM accumulated by lentils were also linearly associated with the amount of iPAR. Linear relationships have been observed in many crops (potato, chickpea, cowpea, maize, soybean, wheat) by various researchers [8,13,14,40,41].

As our data showed, the effect of fertilization on iPAR values depended on meteorological conditions and acted through LAI development and AGDM accumulation. Fertilization was not the main factor determining the variation in iPAR data and was responsible only for 1.6–6.7% of iPAR variation. According to the multiple linear regression model, it was found that seed yield significantly depended on LAI, AGDM, and iPAR at different growth stages, at BBCH 51, BBCH 65, and BBCH 69, respectively, 40.2, 27.4, and 52.4%.

The efficiency of nitrogen use in a crop is defined as nitrogen use efficiency (NUE). To preserve the environment and improve sustainable and productive agriculture, limiting the use of N fertilizers and increasing NUE are important factors [42]. Applying lower N rates results in higher NUE compared to higher fertilization [43,44]. NUE varies between cultivars, and the relationship between cultivar and N level shows that cultivars with higher NUE under high N rates do not always maintain high NUE under lower N supply [45]. In our experiment, differences in both NUE yield and NUE protein yield between varieties were also determined. Averaged across varieties, the highest values of the indices were obtained under lower N rates (NUE yield—N30 and N45, and NUE protein yield—N15 and N30).

## 4. Material and Methods

### 4.1. Location of the Study

A field experiment was conducted at the Institute of Agriculture, Lithuanian Research Centre for Agriculture and Forestry in Central Lithuania (55°23′50″ N and 23°51′40″ E) during 2015–2017. Soil samples for agrochemical characteristics assessment were taken from the 0–20 cm layer each year before pea sowing. The available phosphorus, available potassium, humus, and pH in the experimental plots ranged within 84–150 mg kg^−1^, 140–186 mg kg^−1^, 1.5–2.2%, 5.4–7.2, respectively, according to the A-L method, A-L method [46], Tyurin method [47] and the potentiometric method, respectively. The mineral nitrogen before sowing in the 0–40 cm soil layer ranged from 43 to 59 kg ha^−1^ (as the sum of N-NO_3_ and N-NH_4_, N-NO_3_—ionometrically, N-NH_4_—spectrophotometrically.)

### 4.2. Soil and Meteorological Conditions

**Figure 6:**
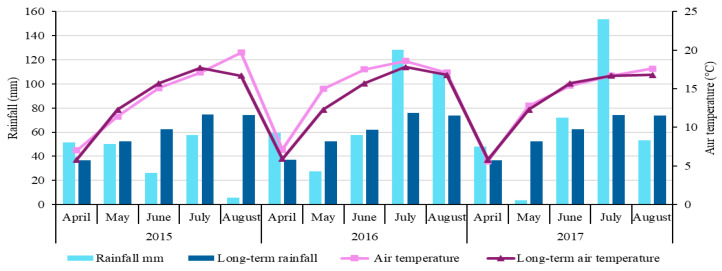
The distribution of rainfall and temperature during the growing seasons.

The weather conditions were given by the data of the meteorological station at the Dotnuva meteorological station. Rainfall and mean air temperature at the experimental site over the three growing seasons are provided in Figure 6.

**Figure 7:**
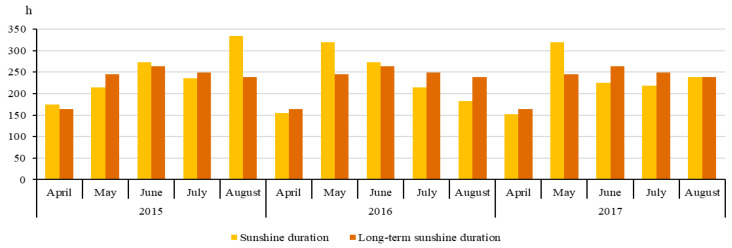
Sunshine duration during the growing seasons.

The precipitation amounts in the 2015, 2016, and 2017 growing seasons were 191 mm, 382 mm, and 229 mm, respectively. Sunshine duration during the growing seasons in 2015–2017 is provided in Figure 7. In 2015, the highest sunshine duration was in August—334.5 h, while in 2016 and 2017, the longest sunshine duration was in May, 320.2 and 319.4 h, respectively.

### 4.3. Experimental Design and Crop Management Practices

The experiment was arranged in a factorial randomized block design consisting of three levels of the first factor (variety) and eight levels of the second factor (fertilization NPK) in various combinations with a total of 24 treatments replicated four times. Detailed treatments are presented in Table 6. All fertilizers, N (as ammonium nitrate), P (as granular superphosphate), and K (as potassium chloride), were applied before sowing and incorporated into the soil. Treatment six was additionally fertilized with N 15 during the stem growth stage (BBCH 30–49). The plot size was 15.0 m^2^ (1.5 m × 10.0 m).

The pea varieties grown in the experiment were semi-leafless—Ieva DS, Simona, and Respect. The pea variety Respect is medium-early, created in Denmark. Simona previously bred a Lithuanian variety of medium height. Ieva DS is also a variety created by Lithuanian breeders, included in the National List of Plant Varieties at the beginning of the research; its productivity potential has not been studied at different nutritional levels.

According to the experimental plan, fertilizers were applied before sowing and incorporated into the soil. The pre-crop was spring barley (*Hordeum vulgare* L.) in all experimental years. Peas were sown in the second ten-day period of April with a small-sized precision sowing machine. Seed rate was 1.2 million viable seeds per hectare, with an 8 cm distance in the rows and a 13 cm distance between rows. The seed sowing depth was 4–5 cm.

The herbicide Fenix (a.i. aclonifen, 600 g L^−1^) (3.0 L ha^−1^) was sprayed after sowing, each year. Chemical insect control was performed using the following products at BBCH 14–16: Fastac 50 (a.i. alfa-cipermetrin 50 g L^−1^) (0.20 L ha^−1^) in 2014 and 2015 and Decis Mega 50 EW (i.e., deltametrin 50 g L^−1^) (0.15 L ha^−1^) in 2016 and 2017.

### 4.4. Estimation of Growth Parameters and Intercepted Solar Radiation

All measurements were made on typical sunny days, three times per growing season—at inflorescence emergence (BBCH 51), at the full flowering (BBCH 65), and at the end of flowering (BBCH 69). (BBCH = biologische, bundesanstalt, bundessortenamt, and chemical).

The leaf area index (LAI) and intercepted photosynthetic active radiation (iPAR) were measured using a SunScan SSI (Delta T Devices Ltd., Cambridge, UK). The instrument consists of a handheld device with a 1-metre-long probe with 64 diodes that measure PAR intensities and a separate station that measures the PAR incidence on the canopy top. The data were obtained using a 1-metre-long sensor placed at the middle of the pea inter-rows parallel to the direction of the rows, at the soil surface. The intercepted PAR was calculated as the ratio of the difference between incident and transmitted radiations to incident radiation [16].

Aboveground dry biomass (AGDM) was measured at BBCH 51, BBCH 65, and BBCH 69 GS using a destructive sampling method. At each sample collection, all the plants from an area of 0.25 m^2^, from the inner rows of plots, were cut aboveground level, chopped, and oven-dried at 80 °C for 48 h and then the dry samples were weighed.

### 4.5. Nitrogen Efficiency Estimate

The following agronomic nitrogen use efficiency (NUE, kg kg^−1^ N) was calculated for each treatment:

NUE_YIELD_ = (Yield _N_ − Yield _N0_)/N_x_ (1)

where Yield _N_ is grain yield (kg ha^−1^) from nitrogen (N) fertilized treatments, Yield _N0_ is grain yield in the unfertilized treatment, and N_x_ is nitrogen input (kg N ha^−1^, N_15_, N_30_, N_45_, N_60_).

Nitrogen uptake efficiency (NUE _PROT YIELD_) or variation in grain protein yield (kg kg^−1^ N) was calculated according to the following formula:

NUE _PROT YIELD_ = (PROT YIELD_N_ − PROT YIELD_N0_)/N_x_ (2)

where PROT YIELD_N_ is grain protein yield (kg kg^−1^ N) in N fertilized treatments, PROT YIELD_N0_ is grain protein yield in the control treatment, N_x_ is N rate (kg N ha^−1^, N_15_, N_30_, N_45_, N_60_).

### 4.6. Statistical Analysis

A three-way ANOVA was used to determine the effects of growth stage, variety, and fertilization level on the growth parameters and intercepted PAR. The least significant difference (LSD) was calculated with Fisher’s test at the probability levels *p* ≤ 0.05 and *p* ≤ 0.01. The homogeneity and normality of data were verified using Bartlett’s test before conducting ANOVA. Standard statistical procedures were used for calculating simple correlation coefficients. The statistical analysis was performed using STAT ENG software from the statistical data processing package Selekcija (version 6).

## 5. Conclusions

Nitrogen (N) fertilization positively influenced LAI, but LAI values significantly increased only under N45 and N60, and splitted N30 (N15 + N15) applications, compared to treatment N0P40K80. In the dry 2015 and the optimal moisture 2016, N30, N45, and N60 rates significantly increased AGDM. The influence of fertilization on iPAR varied between experimental years, and it was strongest in the dry 2015, when applying N15 + 15 and N60 fertilization significantly increased iPAR compared to the control. According to LAI and iPAR data, pea varieties were ranked in descending order: Simona, Ieva DS, and Respect. The data averaged across varieties showed that LAI significantly (*p* ≤ 0.01) and positively correlated with AGDM and iPAR in all cases, but the relationship weakened as peas reached later growth stages. The multiple linear regression model revealed that relationship between seed yield and LAI, PAR, and AGDM was significant and explained 40.2%, 27.4%, and 52.4% of the yield variation, respectively, at BBCH 51, BBCH 65, and BBCH 69.

These results provide valuable knowledge and will be useful for researchers in developing new cultivation methodologies to achieve higher semi-leafless pea productivity by applying different combinations of nutrition and new varieties that influence crop leaf area, dry matter production, and improve the amount of intercepted PAR and resource efficiency.

## Figures and Tables

**Table 1:** The contribution (% of the sum squares) of growth stage (GS), variety, and fertilization to total variance in leaf area index (LAI), aboveground dry mass (AGDM), and intercepted photosynthetically active radiation (iPAR) of pea.

Factors	DF	2015	2016	2017
LAI				
GS (A)	2	52.3 **	61.2 **	12.7 **
Variety (B)	2	29.8 **	13.3 **	28.0 **
Fertilization (C)	6	0.6 **	3.8 **	11.4 **
AGDM				
GS (A)	2	58.1 **	75.5 **	47.4 **
Variety (B)	2	14.1 **	2.7 **	5.3 **
Fertilization (C)	6	4.7 **	3.4 **	0.8
iPAR				
GS (A)	2	43.7 **	72.4 **	3.6 **
Variety (B)	2	22.4 **	8.4 **	16.8 **
Fertilization (C)	6	2.4 **	2.1 **	6.9 **

** significant at *p* ≤ 0.01.

**Table 2:** Correlation matrix of LAI, AGDM, and iPAR across varieties at different growth stages (average data 2015–2017).

Indices	Mean Values	AGDM1	iPAR1	AGDM2	iPAR2	AGDM3	iPAR3
LAI1	3.04	0.857 **	0.924 **				
AGDM1	135.6	1.00	0.811 **				
iPAR1	82.16		1.00				
LAI2	3.52			0.692 **	0.899 **		
AGDM2	241.35			1.00	0.605 **		
iPAR2	87.11				1.00		
LAI3	4.67					0.368 **	0.542 **
AGDM3	344.76					1.00	0.366 **
iPAR3	80.22						1.00

LAI—leaf area index, AGDM—aboveground dry mass, iPAR—intercepted photosynthetically active radiation, 1, 2, and 3—indices measured at BBCH 51, BBCH 65, and BBCH 69. **—relationship is significant at *p* ≤ 0.01.

**Table 3:** Correlation coefficients between the seed yield, seed quality, and traits studied of pea.

Variety	Indices	LAI1	LAI2	LAI3	AGDM1	AGDM2	AGDM3	iPAR1	iPAR2	iPAR3
Ieva DS	SY	0.175	0.444 *	0.669 **	0.455 *	0.468 *	0.440 *	0.014	0.291	0.119
	PC	0.607 **	0.648 **	0.310	0.779 **	0.699 **	0.492 *	0.383	0.543 *	0.149
	TSW	0.482 *	0.225	−0.273	0.249	0.178	0.115	0.628 **	0.367	0.637 **
Simona	SY	0.651 **	0.703 **	0.728 **	0.664 **	0.513 *	0.642 **	0.645 **	0.681 **	0.332
	PC	0.721 **	0.694 **	0.185	0.685 **	0.377	0.631 **	0.614 **	0.504 *	0.020
	TSW	−0.149	−0.03	−0.664 **	0.002	−0.058	−0.001	−0.339	−0.176	−0.059
Respect	SY	0.821 **	0.797 **	0.736 **	0.763 **	0.787 **	0.712 **	0.625 **	0.537 **	0.721 **
	PC	−0.845 **	−0.793 **	−0.675 **	−0.787 **	−0.702 **	−0.641 **	−0.691 **	−0.541 **	−0.680 **
	TSW	0.838 **	0.855 **	0.846 **	0.867 **	0.860 **	0.902 **	0.869 **	0.849 **	0.870 **
Mean across varieties										
	SY	0.504 **	0.536 **	0.578 **	0.585 **	0.360 **	0.440 **	0.392 **	0.472 **	0.027
	PC	0.258 *	0.315 **	0.324 **	0.057	0.038	0.017	0.178	0.234	0.220
	TSW	0.263 *	0.269 *	−0.028	0.256 *	0.188	0.206	0.232	0.251 *	0.343 **

LAI—leaf area index, AGDM—aboveground dry mass, iPAR—intercepted photosynthetically active radiation, 1, 2, and 3—traits studied of pea at BBCH 51, BBCH 65, and BBCH 69; SY—seed yield (t ha ^−1^), PC—protein content (%), TSW—thousand seed weight (g). * and **—relationship is significant at *p* ≤ 0.05 and at *p* ≤ 0.01, respectively.

**Table 4:** The relationship between intercepted iPAR (y_1_) and leaf area index (LAI) (x), and between aboveground dry mass (AGDM) (y_2_) and intercepted iPAR (z) (average data 2015–2017).

Growth Stages	Variety	iPAR (y_1_) Relation with LAI (x)		AGDM (y_2_) Relation with iPAR (z)	
		Regression equation	R^2^	Regression equation	R^2^
BBCH 51	Ieva DS	y_1_ = 64.634 + 5.721x	0.895 **	y_2_ = 72.833 + 0.078z	0.651 **
	Simona	y_1_ = 66.031 + 5.341x	0.912 **	y_2_ = 69.642 + 0.124z	0.736 **
	Respect	y_1_ = 63.676 + 6.095x	0.778 **	y_2_ = 60.971 + 0.117z	0.876 **
BBCH 65	Ieva DS	y_1_ = 69.884 + 5.570x	0.933 **	y_2_ = 75.322 + 0.064 z	0.490 **
	Simona	y_1_ = 66.084 + 5.875x	0.762 **	y_2_ = 66.069 + 0.085z	0.307 **
	Respect	y_1_ = 59.791 + 7.784x	0.803 **	y_2_ = 59.530 + 0.099z	0.702 **
BBCH 69	Ieva DS	y_1_ = 75.757 + 3.210x	0.220 *	y_2_ = 80.854 + 0.031z	0.194 *
	Simona	y_1_ = 93.455 + 0.451x	0.210 *	y_2_ = 95.263 + 0.001z	0.217 *
	Respect	y_1_ = 75.808 + 3.898x	0.869 **	y_2_ = 80.059 + 0.027z	0.711 **
Data averaged across varieties					
BBCH 51		y_1_ = 64.295 + 5.852x	0.854 **	y_2_ = 68.179 + 0.103z	0.658 **
BBCH 65		y_1_ = 64.327 + 6.459x	0.808 **	y_2_ = 69.871 + 0.071z	0.366 **
BBCH 69		y_1_ = 81.217 + 2.459x	0.294 **	y_2_ = 84.980 + 0.021z	0.134 **

* and **—relationship is significant at *p* ≤ 0.05 and at *p* ≤ 0.01, respectively.

**Table 5:** Correlation coefficient (R) of the multiple correlation between seed yield and LAI, SM, iPAR, and LAI and meteorological conditions across varieties at different growth stages (average data 2015–2017).

Parameters	BBCH 51		BBCH 65		BBCH 69	
(y)	R	F_fact._	R	F_fact._	R	F_fact._
Relation between SY and LAI, iPAR and AGDM						
SY	0.627	12.71 **	0.536	7.93 **	0.745	24.49 **
Relation between LAI, AGDM, and precipitation and accumulated growing degree days >10 °C						
LAI	0.913	150.03 **	0.914	152.13 **	0.539	12.26 **
AGDM	0.953	293.32 **	0.721	32.47 **	0.774	44.73 **

Multi regression equation y = a + bx1 + cx2 + dx3, where y—seed yield (SY), x1—leaf area index (LAI), x2—intercepted photosynthetically active radiation (iPAR), x3—aboveground dry mass (AGDM), or equation y = a + bx1 + cx2, where y—LAI or AGDM, x1—precipitation, x2—accumulated growing degree days > 10 °C. **—relationship between indices is significant at *p* ≤ 0.01.

**Table 6:** Treatments details of the experiment.

Variety (Factor 1)	Fertilization (Factor 2)
1. Ieva DS	1. NPK 0:0:0
2. Simona	2. 0:40:80
3. Respect	3. 15:40:80
	4. 30:40:80
	5. 45:40:80
	6. 15 + 15:40:80
	7. 60:40:80

## Data Availability

Data are contained within the article.

## Abbreviations

The following abbreviations are used in this manuscript:

LAI	Leaf area index
AGDM	Aboveground dry mass
iPAR	Intercepted photosynthetically active radiation
GS	Growth stage
SY	Seed yield
PC	Protein content
TSW	Thousand seed weight
NUE	Nitrogen use efficiency
BBCH	Biologische, bundesanstalt, bundessortenamt, and chemical
